# Geographic and socioeconomic differences in potentially inappropriate medication among older adults – applying a simplified analysis of individual heterogeneity and discriminatory accuracy (AIHDA) for basic comparisons of healthcare quality

**DOI:** 10.1186/s12913-025-13335-y

**Published:** 2025-08-28

**Authors:** Johan Öberg, Kani Khalaf, Raquel Perez-Vicente, Kristina Johnell, Johan Fastbom, Juan Merlo

**Affiliations:** 1 Unit for Social Epidemiology, Faculty of Medicine, Lund University, Malmö, Sweden; 2 Department of Health and Medical Care Management, Region Skåne, Malmö, Sweden; 3 Department of Medical Epidemiology and Biostatistics, Karolinska Institutet, Stockholm, Sweden; 4 Aging Research Center, Karolinska Institutet, Stockholm, Sweden; 5 Centre for Primary Health Care Research, Region Skåne, Malmö, Sweden

**Keywords:** MeSH, Social epidemiology, Health care quality assessment, Pharmacoepidemiology, Health services evaluation, Epidemiological methods

## Abstract

**Background:**

Monitoring of healthcare quality is typically focused on differences between group averages in relation to a desirable benchmark. However, we need to consider (*i*) the existence of interconnected socioeconomic axes of inequality like age, sex, income, and country of birth and (*ii*) individual heterogeneity around group averages. Additionally, (*iii*) we need clear criteria to quantify group differences. By applying the framework *analysis of individual heterogeneity and discriminatory accuracy* (AIHDA) on an established quality indicator (potentially inappropriate medication (PIM)), we illustrate how to achieve these improvements and how to avoid both unnecessary group stigmatization and false expectations.

**Methods:**

We analyzed 731,339 individuals, *≥* 75-year-old belonging to 36 socioeconomic strata defined by the intersection of age, sex, income, and country of birth, who were alive and residing in the 21 regions Swedish during 2011. We calculated PIM prevalences and evaluate the discriminatory accuracy (DA) of the socioeconomic and geographical group differences using the area under the ROC curve (AUC). The benchmark value was defined as a prevalence of 19%.

**Results:**

In Sweden, the prevalence of PIM was 24% among *≥* 75-year-olds and regionally it ranged between 21% and 27%. Immigrant 80–84-year-old women with low income had the highest prevalence (29%). All strata including women had higher prevalence than those including men. However, the regional (AUC = 0.520) and socioeconomic (AUC = 0.544) differences were very small. For instance, in the five socioeconomic strata with the lowest prevalence there were about 8,000 more cases of PIM than in the five strata with the highest prevalence of PIM.

**Conclusion:**

The prevalence of PIM was higher than the desired benchmark value. There were disparities between group averages, but overall, the regional and socioeconomic differences were very small as informed by their low AUC values. Therefore, interventions to reduce PIM in Sweden should be universal rather than only targeted at the regions and socioeconomic strata with the highest PIM prevalence.

**Supplementary Information:**

The online version contains supplementary material available at 10.1186/s12913-025-13335-y.

## Introduction

In Sweden as in many other countries, high-quality healthcare is defined as safe, accessible, delivered in time, health promoting, as well as knowledge- and evidence-based [1, 2]. In addition, to be effective and efficient, good healthcare must be equitable. That is, on equal terms and according to patients’ needs. To establish that Swedish healthcare is delivered based on these principles, The Swedish National Board of Health and Welfare (NBHW), the Swedish Association of Local Authorities and Regions (SALAR), a multitude of Swedish National quality registers and the 21 administrative regions in Sweden evaluate healthcare by defined quality indicators [3] which can be compared to benchmark values. These quality indicators are regularly monitored by means of simplified comparisons between hospitals and administrative regions, such as in reports of *Open comparisons of healthcare performance* [4]. There are good reasons to monitor healthcare [5], however current comparisons could be improved for at least three reasons.

*First*, the existence of healthcare inequities conditioned by interconnected demographic and socioeconomic factors (in the rest of this paper the term socioeconomic also includes demographic dimensions) like age, sex, income and country of birth have been shown in many studies, however today’s official monitoring is focused on geographical disparities rather than socioeconomic factors. Monitoring both geographic and interconnected socioeconomic inequalities is necessary to improve the evaluation of healthcare performance [6].

*Second*, healthcare quality monitoring is often based on the analysis and interpretation of differences between group averages, neglecting individual heterogeneity around group averages [7, 8]. This is an important but often ignored aspect epidemiology and healthcare evaluation [7–10].

*Third*, we need clear criteria for quantifying the size of the group differences. In comparisons and evaluations of healthcare quality, this is seldom clearly stated and therefore the size of group differences is hard to evaluate. To determine whether there are relevant regional differences in a quality indicator, it is important to look beyond mere statistical significance. Small and meaningless differences between group averages can appear statistically significant if the sample size is large enough. Additionally, measurable differences between group averages can coexist with substantial individual heterogeneity within groups, which may diminish the relevance of average differences.

In this study, we apply the method analysis of individual heterogeneity and discriminatory accuracy (AIHDA), which is a simple but innovative analytical framework that has the potential to address the weaknesses mentioned above and to improve the quantitative evaluation of health care quality [11]. As discussed previously [8, 11, 12], when applying AIHDA, we do not regard differences between group averages and individual patient differences as unrelated phenomena. Instead, we adopt a conceptual multilevel perspective that integrates both types of differences (within group differences and between group differences). From this viewpoint, we do not measure socioeconomic and geographical group differences by simply comparing group averages. For binary patient outcomes (such as most quality indicators), we can evaluate how well group-level information can distinguish individuals with the outcome of interest from those without it, and thus evaluate to what extent the differences are related to group membership. By quantifying this procedure via a ROC-curve and its AUC-value, we can assess group differences and compare the relevance of different grouping criteria (e.g., socioeconomic strata versus geographical regions). AIHDA can be applied using traditional regression [13] or more advanced multilevel models, referred to as multilevel-AIHDA (MAIHDA) [6, 9, 10, 14]. While we mainly recommend multilevel models for healthcare monitoring [15], AIHDA provides a simplified but valid alternative for routine monitoring of quality indicators. Motivated by the above background and following a previous publication [11] we aim to further illustrate how the AIHDA approach can be applied in official comparisons and reports evaluating healthcare quality with focus on geographical and socioeconomic inequalities.

In this study we analyze an established quality indicator denominated *potentially inappropriate medication (PIM) among individuals 75 years and older* [16] and selected all people 75 years and older who were alive and residing in Sweden in 2011. PIM are pharmaceuticals that in many cases pose more risks than benefits, especially when safer alternatives exist. In the evaluation of healthcare quality, PIM serves as a quality indicator because prescribing fewer PIMs is associated with better, safer, and more effective care for patients, particularly in vulnerable populations like the elderly. For instance, glibenklamid (also known as glyburide) is included in the list of PIM for older adults provided by the NBHW in Sweden. This medication is noted for its high risk of causing hypoglycemia among older adults, particularly those with reduced kidney function. Because of this, safer alternatives are often recommended for managing diabetes in older adults. Some patients may benefit from medications classified as PIM. Consequently, the benchmark value for this indicator is not set at zero, but rather aims to minimize PIM use on a population level. This approach acknowledges the individual therapeutic needs and circumstances that may justify PIM prescriptions while striving for overall reduction in their use. The quality indicator helps healthcare providers to monitor medication safety and overall treatment quality. The definition of PIM used in this study is aligned with NBHW’s definition which can be found in Table 1.

**Table 1:** Potentially inappropriate medications, National board of health and welfare in Sweden (2024)

Group	ATC	Drug
Psycholeptics	N05BA01	diazepam
	N05CD02	nitrazepam
	N05CD03	flunitrazepam
	N05CM06	propiomazine
	N05AA02	levomepromazin
	N05AB04	prochlorperazine
	N05AF03	chlorprothixene
	N05AH02	clozapine
	N05BB01	hydroxizine
Analgesics	N02AX02	tramadol
	N02AJ06	codeine and paracetamol
	N02AJ09	codeine and other non-opioid analgesics
Couch and cold preparations	R05DA04	codeine
Drugs used in diabetes	A10BB01	glibenclamide
Drugs for functional gastrointestinal disorders	A03AB	glycopyrronium
	A03BA	atropine, hyoscyamine
	A03BB	butylscopolamine, methylscopolamine
Antiemetics and antinauseants	A04AD	scopolamine
Cardiac therapy	C01BA	disopyramide
Urologicals	G04BD exclusvive G04BD12	oxybutynin, tolterodine, solifenacin, darifenacin, fesoterodine
Muscle relaxants	M03BC01	orphenadrine (citrate)
	M03BC51	orphenadrine, combinations
Opioids in combination with spasmolytikum	N02AG	morphine, ketobemidone, hydromorphone in combination with antispasmodics
Anti-parkinson drugs	N04A	trihexyfenidyl, biperiden
Psycoanaleptics	N06AA	clomipramine, amitriptyline, nortriptyline, maprotiline
Antihistamines for systemic use, some (1st generationen)	R06AA02	diphenhydramine
	R06AA04	clemastine
	R06AB	chlorphenamine, dexchlorpheniramine
	R06AD	alimemazine, promethazine, thiethylperazine
	R06AE05	meclozine
	R06AX02	cyproheptadine

## Population and methods

### Databases

Our investigation is based on a database created by record-linkage of several registers with national coverage, including the Swedish Total Population Register (TPR) [17] and the Longitudinal Integration Database for Health Insurance and Labour Market Studies (LISA) [18], administrated by Statistics Sweden (SCB),, the Cause of Death Register [19] and the Swedish Prescribed Drug Register (SPDR) [20], administrated by NBHW. The SPDR contains information on all prescription drug dispensed by Swedish pharmacies. However, drug dispensations for inpatient care within hospitals and nursing homes from drug storerooms are not included. An overview of information retrieved from each register can be found in Table 2.

**Table 2:** Overview of retrieved information from Swedish National registers

Register	Information retrieved
Swedish Total Population Register (TPR)	Study population: Older adults (75 years and older) residing in Sweden 31 December 2010
Cause of Death Register (CDR)	Exclusion of deceased
Longitudinal Integration Database for Health Insurance and Labour Market Studies (LISA)	Sex, age, household income, country of birth
Swedish Prescribed Drug Register (SPDR)	Dispensations of PIM

To maintain confidentiality, the registers were linked using an arbitrary serial number assigned to everyone by Statistics Sweden instead of the Swedish unique personal identification number.

The Regional Ethics Review Board in Southern Sweden (ID 2014/856) and the data safety committees from the NBHW (ID 9542/2015) and from Statistics Sweden (ID 231424/878144-5) approved the construction of the database.

### Study population

**Fig. 1:**
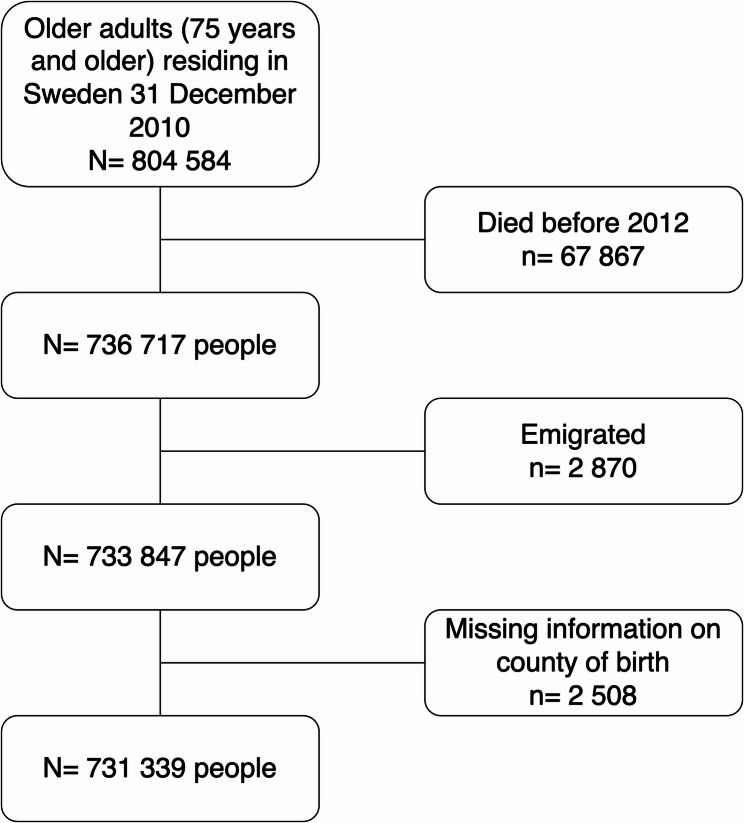
Study population flow-chart

We defined the study population using the same criteria as applied by NBHW for the quality indicator *PIM among older people* [16]. The initial population included all 804,584 individuals aged 75 years and older, who were residing in Sweden by 31 December 2010. We followed those individuals for 12 months and we excluded individuals who died (*N* = 67,867), emigrated (*N* = 2,870) or had missing information on country of birth (*N* = 2,508). The final study population included 731,339 individuals (91% of the original study population). See Fig. 1.

### Assessment of variables

#### Outcome variable: Potentially inappropriate medication (PIM) among older people

There are many definitions of PIM among older people, such as the definitions used by The American Geriatrics Society (AGS), the Beers Criteria [21], the EU [7]-PIM [22], the FORTA PIM-list [23] and the PRISCUS PIM-list [24]. However, in our study we choose to use the definition by the NBHW in Sweden (Table 1) because it is important to adapt the definitions according to the local context [24]. At the individual level, the outcome in our study was having at least one dispensation during 2011 of any of the drugs classified as PIM by the NBHW [15]. PIM can only be prescribed via medical doctors and are dispensed via Swedish pharmacies.

#### Benchmark value

At the aggregated level there is not an officially predefined benchmark value, but the NBHW indicates that the percentage of patients with PIM should be as low as possible [25]. For the purpose of our study, we set the benchmark value equal to 19% which is the lowest observed prevalence in our study and corresponds with the stratum of 75–79-year-old native men with high income. Our reasoning is to use a realistic benchmark value which has been observed in a real-world setting. It is not possible to determine the optimal level of PIM in the study population, since medication classified as PIM is appropriate in some instances on an individual level where estimated benefits outweigh the risks. According to NBHW, PIM should be as low as possible [16].

#### Administrative regions

We classified the individuals into the 21 administrative regions of Sweden according to their legal residence address by 31 December 2010. Administrative regions in Sweden form autonomous political units and act as principals for health care delivery, which may condition regional inequalities in the risk of PIM due to specific healthcare policies as well as differences in therapeutical practices and resources.

#### Individual variables

We categorized *age* into three groups: 75–79, 80–84, 85 years and older. *Sex* was defined as men or women according to the registered information. We categorized the patients according to their *country of birth* into native (i.e., born in Sweden) or not (i.e., immigrant) according to the Swedish population register. To operationalize socioeconomic position, we use *individualised disposable family income* for the years 2000, 2005 and 2010. We calculated tertiles of income based on an established procedure utilized in previous studies within our research database, see elsewhere for details [6, 11].

#### Socioeconomic multicategorical variable

Finally, we constructed a socioeconomic multicategorical variable consisting of all possible combinations of the categories of the four socioeconomic variables age, sex, income and country of birth and thus resulting in 36 *socioeconomic strata*. This multicategorical variable allows a better mapping of the demographic and socioeconomic distribution of risk.

### Statistical and epidemiological analysis for the AIHDA-framework

For evaluating geographical and socioeconomic differences, the AIHDA-framework applies four consecutive steps described in detail elsewhere [9, 11].

#### 1^st^ step, identifying the quality indicator and the benchmark or target average value

As informed before, the quality indicator in focus was PIM among older adults and the benchmark value was set to 19%.

#### 2^nd^ step, visualizing and evaluating differences between group averages

For this purpose, we calculate the crude percentage of patients with PIM and their 95% confidence intervals (CIs), overall and in each administrative region as well as in each multicategorical socioeconomic stratum. This step provides an improved mapping of risk (i.e., prevalence) in the form of tables and figures (e.g., caterpillar plots). This step corresponds to the traditional analysis based on group averages, where we identify if specific groups have a noticeable higher or lower prevalence of PIM compared to the benchmark value.

#### 3^rd^ step, quantifying the size of the group differences using a measure of discriminatory accuracy (DA) like the area under the receiver operating characteristic curve (AUC)

In AIHDA for a binary quality indicator, group differences are evaluated as the accuracy of the socioeconomic and geographical information for discriminating individual cases from non-cases of PIM. For this purpose, we used regression models to calculate the predicted probabilities and the AUC with 95% CIs as a measure of DA. We performed two main regression models. *Model 1* estimates differences between administrative regions by including only the variable with the 21 administrative regions and *Model 2* estimates socioeconomic differences by including the socioeconomic multicategorical variable only. Since the prevalence of the PIM outcome was not extreme, we applied Cox proportional hazards regression models with a constant follow-up time equal to 1 [25]. In the socioeconomical analysis the strata of native 75–79-year-old men with high income served as reference category, i.e. the stratum which prevalence of PIM serves as our benchmark value.

In the geographical analysis we used “Region Skåne” as the reference in the comparisons. This decision was arbitrary and based on the affiliation of the research team to Region Skåne.

The AUC is obtained by plotting the true-positive fraction against the false-positive fraction for different thresholds of the predicted probability of PIM based on the geographical and/or the socioeconomic information, i.e. all observations within a certain socioeconomic strata or region will have the same predicted probability. The value of the AUC ranges from 0.5 to 1, with 0.5 indicating no discriminatory accuracy and 1 representing perfect discrimination. Using the criteria proposed by Hosmer and Lemeshow [26], we classified DA and thereby, the group differences as very small (0.5 ≤ AUC ≤ 0.6), small (0.6 < AUC ≤ 0.7), large (0.7 < AUC ≤ 0.8), or very large (AUC > 0.8), as discussed in previous publications [9, 11].

In addition to obtaining the prediction for the calculation of the AUC we also obtained prevalence ratios (PRs) and their robust 95% confidence intervals (CIs) [25]. The values can also be used in the 2nd step described above for estimating and visualizing the groups’ relative differences comparing to a reference group.

##### Number of PIM cases and non-cases in regions and socioeconomic strata

To further understand the concept of DA we notice the size of each stratum as how cases and non-cases of PIM were distributed across administrative regions and the socioeconomic strata. We can calculate the number of PIM cases in the five groups with the lowest group average risk (expressing “false negative”) and the number of PIM cases in the five groups with the highest group average risk (expressing “true positive”). Similarly, we can calculate the number of “false positive” as the number of individuals without PIM in the five groups with the highest group average risk. Measuring geographical and socioeconomic differences to evaluate healthcare quality is analogous to performing a diagnostic test [7, 27]. That is, we use geographical and socioeconomic differences to classify individuals in relation to the presence of absence of PIM. From this perspective we do not want to unnecessarily treat false positive individuals or incorrectly deny treatment of false negative patients. In our case, we want to know the accuracy of open geographical and socioeconomic comparisons for discriminating individuals with PIM from those without PIM.

#### 4^th^ step, interpretation of the results

Finally, in the 4th step we proceed to the interpretation of the results by evaluating DA of the geographical and socioeconomical information in relation to the benchmark value. We aim to use this information to suggest possible interventions to decrease the risk of PIM. Following the studies referred earlier [9, 11], we applied a practical framework for evaluating geographical and socioeconomic differences. For this purpose, we need at least two types of information.

*First*, we need to know whether the average value of the quality indicator for the whole population (i.e., percentage of patients with PIM) has reached the predetermined target value *fully* (i.e., *≤* 19% of all patients were exposed to a PIM), *insufficiently* (i.e., 20–21%), or *not at all reached* (i.e., >21%). The qualifications “*not at all reached*” and “*insufficiently reached*” are subjective and arbitrarily chosen by the authors.

*Second*, we need information about the size of the group differences as assessed by the AUC, which expresses the accuracy of the differences between group averages for discriminating patients with the outcome from those without the outcome. These two types of information need to be combined for the final evaluation. Table 3 illustrates these ideas via a simple framework with 12 scenarios that can be used to orient the interpretation of an analysis.

**Table 3:** Framework for evaluating geographical and socioeconomic differences in a specific quality indicator. For this purpose, we need information about the target indicator value. That is, whether the average quality indicator (i.e., percentage of patients with PIM) was “not at all”, “insufficiently” or “fully” reached a predetermined target benchmark level. We also need information on the area under receiver operating characteristics curve (AUC), which expresses the discriminatory accuracy of the group differences. That is, their ability to discriminate patients with the outcome from those without the outcome. Combining this information, we obtain 12 different scenarios for our evaluation

Size of the group differences as estimated by the AUC-value		Target indicator value reached		
		Fully *≤* 19%	Insufficiently 20–21%	Not at all > 21%
Very small	0.5 ≤ AUC ≤ 0.6	A	B	C
Small	0.6 ≤ AUC < 0.7	D	E	F
Large	0.7 ≤ AUC < 0.8	G	H	I
Very large	0.8 ≤ AUC	J	K	L

In the scenarios C, F, I and L the desired target level has not been achieved in the population. In the scenario C the group differences are very small so the conclusion would be that all groups have been treated equally but inappropriately. In scenario L, the group differences are very large, meaning that some groups may have achieved the desired target level while other groups have not, which means that the quality of healthcare in the whole population is unequal and low. Scenario C and L are both undesirable; In scenario L the whole population has not achieved the desired target level and in addition there are inequalities. In scenario C on the other hand, there is equality, but none of the groups have achieve the desirable benchmark value, i.e. equally bad healthcare.

The ideal scenario is however clear. Scenario A, where the desired target level has been fully reached in the population and the group differences are very small. The conclusion would be that all groups have been treated appropriately on equal terms. From this perspective, the quality of the healthcare is high.

As in many other occasion in health care epidemiology the proposed framework is easiest to apply for process quality indicators since they are less susceptible for confounding [27]. For outcome quality indicators the analysis needs to be properly adjusted or interpreted.

Information about DA is crucial for determining if an intervention should be targeted to specific groups or if it should be universal. Observe that in the scenarios A, B and C interventions targeted to specific groups are not justifiable. Rather any intervention should be universal (i.e., directed to the whole country). In scenario A, the reason for the intervention would be to maintain the desirable value of the quality indicator in all groups. In the scenario C, the reason would be to reach this desirable level in all the groups.

In scenario J, K and L, the sizes of the group differences are very large. In scenario J some groups may not have achieved the target level even if the population as a whole has done so. In contrast, in scenario L, some groups may have achieved the target level even if the population, as a whole, has not achieved it. In the scenarios J, K and L, targeted group interventions are justified since the DA is high.

The framework we propose fits well with the concept of proportionate universalism for resource allocation in public health [28, 29]. Based on our analysis we can suggest decision makers to perform targeted, universal or proportionate universal interventions. The AIHDA-approach we apply in this study can be used to make informed decisions regarding the appropriate scale and intensity for a given socioeconomic and/or geographic context [28].

The AIHDA framework we present above is, however, flexible and can be adapted to specific situations. Also, our current study does not cover all possible patterns of results. For instance, a specific group could be an outlier with an extreme undesirable prevalence while the other strata have achieved the benchmark value. In this case a targeted intervention to the outlier group and universal interventions to the other groups could be suitable as the DA is low.

In other words, when planning interventions and assessing healthcare equality it is important to consider the DA, which is related to the size of the different groups and the number of cases in each group. The number of cases is relevant in probabilistic epidemiology as it may concern the uncertainty of the average estimations (i.e. the 95% CI) if the number of cases is low, but it is most pertinent in the real world setting when planning interventions, in terms of false positives and false negatives.

The mapping of each strata’s risk (2nd step in the framework) needs be interpreted together with the DA information in the 3rd step of the AIHDA. While information about each stratum is important for evaluating healthcare equality, it is essential to note that if a change on population level is the aim, only targeting small high-risk strata will not have an impactful effect on the overall population prevalence. This reasoning has been discussed by Rose as the prevention paradox [30]. For planning interventions from a population perspective, it is crucial to consider the overall prevalence of the quality indicator, the specific group prevalences and the DA of the group differences.

## Results

The overall prevalence of PIM in the study population, as shown in Table 4, was 24% (174,311/731,339). The prevalence was higher among women than men as well as among immigrants compared to natives and it also increased with age. Since the benchmark value in this study is set to 19%, the overall population prevalence was not achieved according to the framework in Table 3.

**Table 4:** Prevalence of potential inappropriate medication (PIM), among 731,339 individuals 75 years and older, living in Sweden in 2010, sorted by sex, age, income level and country of birth

		Prevalence of PIM
Total		174 311 (23.8%)
Sex	Men	59 961 (20.6%)
	Women	114 350 (26.0%)
Age	75–79	65 582 (22.6%)
	80–84	55 627 (24.3%)
	≥ 85	53 102 (24.9%)
Income level	Low	62 568 (23.8%)
	Middle	73 109 (24.4%)
	High	38 634 (22.2%)
Country of birth	Native	155 533 (23.7%)
	Immigrant	18 778 (25.2%)

**Fig. 2:**
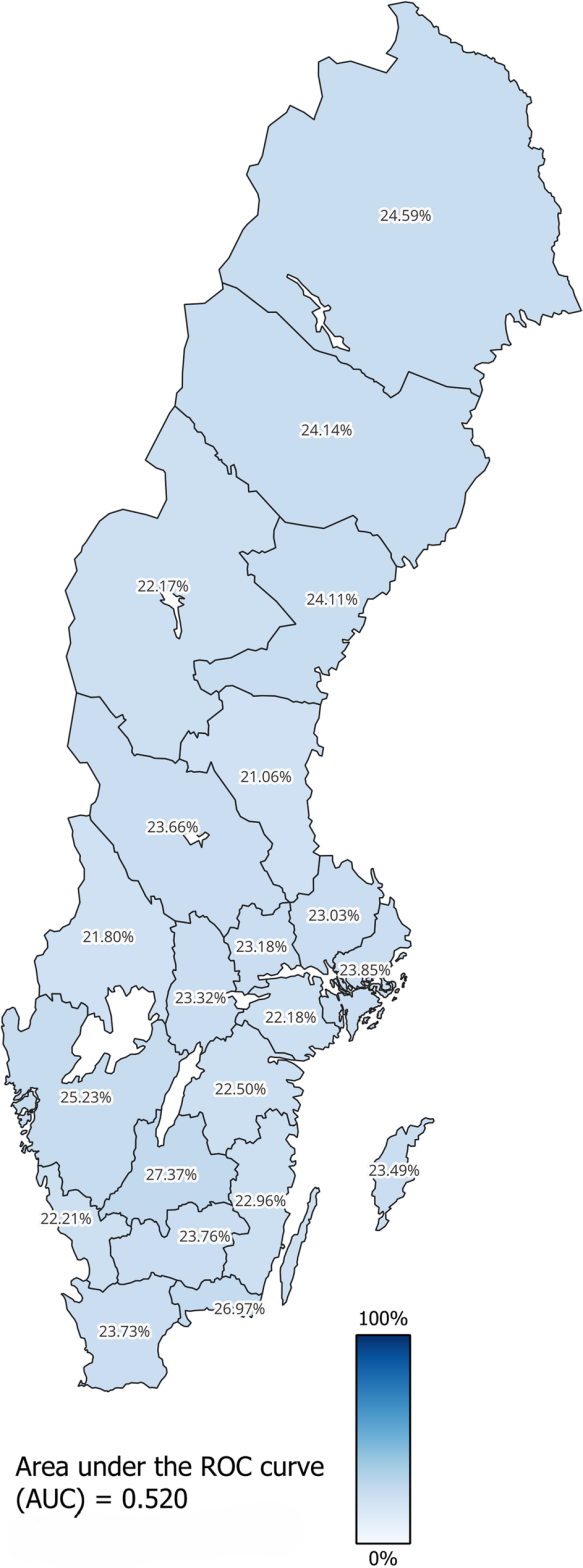
Choropleth map of crude prevalence (Table 5) for use of potentially inappropriate medication (PIM) during the following 12 months among the individuals 75 years and older residing in Sweden by 31 December 2010, grouped into 21 administrative regions, based on Geographic Swedish Data (GSD) overview map [31]

Table 5; Fig. 2 show that the different administrative regions have a PIM prevalence ranging from 21 to 27%. One region *insufficiently reached* the benchmark value, and the rest did not reach the benchmark value *at all*. The AUC value is 0.520 indicating that the accuracy of the geographical differences for discrimination individual with PIM from those without PIM was small.

**Table 5:** Number of individuals (n) with potentially inappropriate medication (PIM), total number of individuals (N) and crude prevalence (P) of PIM during the following 12 months among individuals 75 years and older residing in Sweden by 31 December 2010, grouped into the 21 Swedish administrative regions. Values are numbers, percentages (%) and 95% confidence intervals (CI). For illustrative purposes we specify the number of PIM cases and the number of individuals without PIM for the five strata with highest respectively the lowest PIM risk

Administrative regions	*n*	*N*	*P* (95% CI) %
Gävleborg	*5288*	25 113	21.06 (20.55–21.56)
Värmland	*5662*	25 973	21.80 (21.30–22.30)
Jämtland	*2594*	11 701	22.17 (21.42–22.92)
Södermanland	*5001*	22 547	22.18 (21.64–22.72)
Halland	*5513*	24 822	22.21 (21.69–22.73)
Östergötland	7796	34 650	22.50 (22.06–22.94)
Kalmar	5152	22 437	22.96 (22.41–23.51)
Uppsala	5139	22 313	23.03 (22.48–23.58)
Västmanland	4918	21 220	23.18 (22.61–23.74)
Örebro	5352	22 954	23.32 (22.77–23.86)
Gotland	1173	4994	23.49 (22.31–24.66)
Dalarna	6102	25 788	23.66 (23.14–24.18)
Skåne	22 946	96 714	23.73 (23.46–23.99)
Kronoberg	3888	16 364	23.76 (23.11–24.41)
Stockholm	29 392	123 235	23.85 (23.61–24.09)
Västernorrland	5423	22 489	24.11 (23.55–24.67)
Västerbotten	5140	21 295	24.14 (23.56–24.71)
Norrbotten	5400	21 959	24.59 (24.02–25.16)
Västra Götaland	30 762	121 930	25.23 (24.99–25.47)
Blekinge	3762	13 947	26.97 (26.24–27.71)
Jönköping	7908	28 894	27.37 (26.85–27.88)

Area under the ROC curve (AUC) = 0.520

**Fig. 3:**
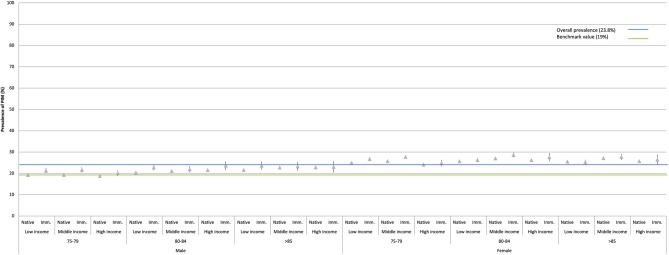
Crude prevalence (P) and 95% confidence intervals (CI) (Table 6) for use of potentially inappropriate medication (PIM) during the following 12 months among the individuals 75 years and older residing in Sweden by 31 December 2010, grouped into 36 socioeconomic strata

Table 6 and Fig. 3 presents socioeconomic differences across the 36 interconnected multicategorical strata. The crude prevalence of PIM for the different strata ranges from 19–29%. Only four socioeconomic strata achieved the benchmark *insufficiently* And the rest of the strata *did not reach* the benchmark value *at all.* All strata including women have higher prevalence of PIM than any of the strata including men. The AUC value is 0.544 which is a slight improvement compared to the model with administrative regions.

**Table 6:** Number of individuals (n) with potential inappropriate medication (PIM), total number of individuals (N) and crude prevalence (P) of PIM during the following 12 months among individuals 75 years and older residing in Sweden by 31 December 2010, grouped into 36 socioeconomic strata. Values are numbers, percentages (%) and 95% confidence intervals (CI). For illustrative purposes we specify the number of PIM cases and the number of individuals without PIM for the five strata with highest respectively the lowest PIM risk

Intersectional socioeconomic strata			n	N	P (95% CI) %		
Men							
75–79	High income	Native	7734	41,092	18.82 (18.44–19.20)		
75–79	Low income	Native	4843	25,143	19.26 (18.77–19.75)		
75–79	Middle income	Native	9490	49,162	19.30 (18.95–19.65)		
75–79	High income	Immigrant	704	3513	20.04 (18.72–21.36)		
80–84	Low income	Native	5732	28,169	20.35 (19.88–20.82)		
80–84	Middle income	Native	7173	33,961	21.12 (20.69–21.56)		
75–79	Low income	Immigrant	1007	4722	21.33 (20.16–22.49)		
80–84	High income	Native	4713	21,851	21.57 (21.02–22.11)		
≥ 85	Low income	Native	5739	26,572	21.60 (21.10-22.09)		
75–79	Middle income	Immigrant	1186	5475	21.66 (20.57–22.75)		
80–84	Middle income	Immigrant	658	2999	21.94 (20.46–23.42)		
≥ 85	Middle income	Native	5421	23,881	22.70 (22.17–23.23)		
≥ 85	High income	Native	3336	14,632	22.80 (22.12–23.48)		
80–84	Low income	Immigrant	773	3386	22.83 (21.42–24.24)		
≥ 85	High income	Immigrant	212	919	23.07 (20.34–25.79)		
≥ 85	Middle income	Immigrant	373	1602	23.28 (21.21–25.35)		
80–84	High income	Immigrant	412	1745	23.61 (21.62–25.60)		
≥ 85	Low income	Immigrant	455	1927	23.61 (21.72–25.51)		
Women							
75–79	High income	Native	8691	36,026	24.12 (23.68–24.57)		
75–79	High income	Immigrant	860	3472	24.77 (23.33–26.21)		
75–79	Low income	Native	10,554	42,278	24.96 (24.55–25.38)		
≥ 85	Low income	Immigrant	1590	6282	25.31 (24.24–26.39)		
≥ 85	Low income	Native	15,614	61,132	25.54 (25.20-25.89)		
80–84	Low income	Native	12,073	47,002	25.69 (25.29–26.08)		
75–79	Middle income	Native	15,823	61,554	25.71 (25.36–26.05)		
≥ 85	High income	Native	4860	18,885	25.73 (25.11–26.36)		
80–84	High income	Native	6137	23,396	26.23 (25.67–26.79)		
80–84	Low income	Immigrant	1960	7464	26.26 (25.26–27.26)		
≥ 85	High income	Immigrant	365	1381	26.43 (24.10-28.76)		
75–79	Low income	Immigrant	2228	8341	26.71 (25.76–27.66)		
80–84	Middle income	Native	13,592	50,284	27.03 (26.64–27.42)		
≥ 85	Middle income	Native	14,008	51,655	27.12 (26.73–27.50)		
80–84	High income	Immigrant	610	2213	27.56 (25.70-29.43)		
≥ 85	Middle income	Immigrant	1129	4081	27.66 (26.29–29.04)		
75–79	Middle income	Immigrant	2462	8881	27.72 (26.79–28.65)		
80–84	Middle income	Immigrant	1794	6261	28.65 (27.53–29.77)		

Area under the ROC curve (AUC) = 0.544

When we assess the DA of the geographical and socioeconomic differences via the AUC-value, it can be concluded that the AUC-value of administrative regions was only 0.520 (Table 5), which according to Table 3 means very small group differences. The socioeconomic information only has an AUC-value of 0.544 (Table 6) which also means very small group differences. Therefore, according to the framework presented in Table 3, we can establish that both the regional and the socioeconomic differences can be categorized within scenario C, i.e. very small group differences with a target indicator that has not been reached.

The low AUC value can be intuitively understood by looking at number of individuals with and without PIM in the regions with high risk and in those with low risk (Tables 7 and 8).

**Table 7:** Number of PIM cases in top five and bottom five regions

No of PIM-cases in the five regions with lowest risk	*N* = 24 058 (“False negatives”)
No of individuals without PIM in the five regions with lowest risk	*N* = 86 098 (“True negatives”)
No of PIM-cases in the five regions with highest risk	*N* = 52 972 (“True positives”)
No of individuals without PIM in the five regions with highest risk	*N* = 155 053 (“False positives”)

**Table 8:** Number of PIM cases in top five and bottom five sociodemographic strata

No of PIM-cases in the five strata with lowest risk	*N* = 28 503 (“False negatives”)
No of individuals without PIM in the five strata with lowest risk	*N* = 118 576 (“True negatives”)
No of PIM-cases in the five strata with highest risk	*N* = 20 003 (“True positives”)
No of individuals without PIM in the five strata with highest risk	*N* = 53 088 (“False positives”)

Table 7 informs that there were 52 972 PIM cases in the five regions with highest risk (i.e., true positives) but also 155 053 individuals without PIM in the same regions (i.e., false positives).

In the Table 8 we see that in the five strata with the lowest risk, there were 28 503 PIM cases (i.e., false negatives), while only 20 003 PIM cases in the five strata with the highest risk (i.e., true positives). That is, there were 8 500 more cases of PIM in the lower than in the higher risk strata. In the five strata with the higher risk, there were 53 088 individuals without PIM (i.e., false positives).

## Discussion

This register study evaluated health care quality with focus on regional and socioeconomic inequalities in PIM among older adults. When doing so, we also aimed to demonstrate how the AIHDA framework can provide improved information when evaluating health care quality with focus on equity. Overall, the aim of the present and previous publications [11, 13, 32] was to introduce the AIHDA methodology using several quality indicators from different healthcare contexts and applying different statical approaches like traditional regression analyses as well as multilevel models [6, 9, 33–35].

### Main findings

We found that the overall prevalence of PIM in our study population was 23.8% and the arbitrary benchmark value set to 19% was not met. The DA for both the regional and the socioeconomic differences was low indicating that those differences were small. So, by applying the framework presented in Table 3, both the regional and the socioeconomic differences could be categorized as scenario C, i.e. very small group differences and a benchmark value which was not met. Therefore, in this scenario we conclude that interventions targeted to specific groups are not justifiable. Rather any intervention should be universal (i.e., directed to the whole country) to reach this benchmark value in all the regions and socioeconomic strata. Nevertheless, a possible universal intervention could be tailored to fit the specific conditions of the regions and socioeconomic strata.

All strata composed by women have higher average risk of PIM than any strata composed by men. Also, higher age, lower income and immigration increases the average risk for PIM to some extent. Traditional interpretation would likely conclude that there were large or considerable differences. However, the AIHDA approach concludes that the socioeconomic and regional differences seem to play a modest role for individual inequalities in PIM among older adults. The AUC-values presented in Tables 5 and 6 provide information about the DA, i.e. to what extent can the group information distinguish between cases and non-cases.

The low DA indicates the existence of many PIM cases in the low-risk groups, as well as many individuals without PIM in the high-risk groups, i.e. false negatives and false positives (Tables 7 and 8).

This fact supports that a potential intervention to prevent PIM should be universal and directed to all regions and socioeconomic strata. Otherwise, the intervention would miss a large portion of the PIM cases. As an example, imagine that we could only intervene or “treat” a single individual without knowing if it has a PIM. The chance of targeting an individual with PIM from our study population would only be marginally larger if we chose an individual from a high-risk strata or region compared to a low-risk strata or region, which the AUC-value shows. Consequently, a practical implication of analysing DA is that it can guide decision-making and planning of healthcare interventions. Fisher et al. makes a distinction between universal policies, proportionate-universal policies and targeted policies [29]. AUC-values close to 0.5 (i.e. very small group differences) suggest that policy interventions need to universal or proportionate-universal and most definitely not targeted. A targeted intervention to decrease PIM, aimed towards the socioeconomic strata with the highest risk of PIM would in fact miss more PIM cases than a targeted intervention aimed towards the five strata with the lowest risk of PIM (Table 8). By adopting the ideas of proportionate universalism [28, 29, 36], information about DA can be used to assess if, and to what extent, interventions should be targeted (i.e. high DA) or universal (i.e. low DA). Interventions should be proportionate in relation to identified risk in terms of prevalences and in relation to DA, in order to prevent under- and overtreatment of groups.

Figure 2 is a representation of the results from Table 5 as a choropleth map. We opted for a map instead of a graph (as Fig. 3 with sociodemographic groups), since geographical differences is natural to interpret via a map (however, an administrative region is both a geographic area and a political unit). There are however challenges with this approach which might overstate group differences between regions [9, 37]. Therefore, choropleth maps should always include a measure of DA, such as the AUC-value, to distinguish the group memberships’ ability to discriminate between cases and non-cases. The colour gradient applied in choropleth maps is important to critically assess to not exaggerate group differences, i.e. in our case we could have used green as the lowest region (prevalence of 21.1%) and red as the highest region (prevalence of 27.4%), resulting in a map filled with both green and red regions. At first glance, this would indicate rather large differences. Instead, we opted to use a mono-coloured gradient reaching from 0 to 100%, which is the hypothetical range of PIM. Still, the geographical representation may create the impression that geographically large administrative regions have more observations and/or cases of PIM (similar to how area represents frequency in a histogram), as they cover extensive land areas. In reality, the most populus regions in Sweden are far from the geographically largest regions. The choropleth map’s design together with the presented AUC-value (0.520) constitutes a graphical presentation which is not exaggerating the group differences.

In our study the socioeconomic differences in PIM prevalence were minor according to the prevalence ratios. As shown in the supplementary material (SM.3), the middle-income group has a slightly higher risk of PIM than the low-income group (prevalence ratio 1.04 (1.03–1.05)). The high-income group has a prevalence ratio of 1.01 (0.99–1.02). In addition, we observed a low AUC value (0.544) indicating that the socioeconomic strata had low discriminatory accuracy. It is important to acknowledge that, if present, the socioeconomic differences in PIM prevalence may arise from a range of underlying mechanisms. These include structural and organizational differences, as well as factors related to clinical decision-making. One potential contributor is unconscious bias among healthcare providers (automatic and unintentional assumptions based on patients’ social or demographic characteristics) which has been shown in previous research to influence treatment decisions [38]. Further research using appropriate casual methods is warranted to investigate factors that may influence the dispensations of PIM.

### How our study agrees/differs from previous research about PIM

The prevalence of PIM among older adults differs greatly between studies, ranging from 8.6–81% [39]. This might be a result of differences in definitions of PIM and study populations [39–46]. The variation in potentially inappropriate prescribing (PIP) across prescribers have been documented to be high [47]. Group differences in PIM have been found in some studies, showing that women, advanced age, lower education, unemployment, suffering from several chronic diseases and being on polypharmacy increase the risk for PIM [40, 44–46]. Increased risk for PIM is also documented among those receiving home care and institutional residents, while differences between socioeconomic groups where greater among people living alone [43]. Our study may underestimate the prevalence of PIM as we did not have information about use of medication from hospitals and nursing home drug storerooms. Those concerns aside, our study builds upon findings from previous research and provides an improved mapping of inequalities based on the AIHDA framework.

### Strengths

Commonly, group averages are exclusively applied for comparisons of healthcare quality. However, this mean-centric approach disregards important information about individual heterogeneity around group averages [4, 48], and thereby missing crucial information which may lead to over-estimating the group memberships’ relevance for the outcome. To solve this challenge, the AIHDA framework includes a measure of DA in the analysis that considers individual heterogeneity in addition to group averages. In AIHDA, information about DA is obtained via the AUC value, which indicates the accuracy of the group-level information for distinguishing between cases and non-cases [9]. The analysis of DA is important in many fields of epidemiology, such as in evaluation of screening, diagnostic and prognostic tests [7, 49–52]. However, the application of DA also benefits the evaluation of healthcare in general [7–10].

As discussed in our previous publications [9, 11] traditional studies exclusively based on differences between group averages implicate several disadvantages as compared to AIHDA. For instance, it is known that epidemiological studies comparing group averages may stigmatize the individuals belonging to groups with “bad” average values and create false expectations for the individuals belonging to groups with “good” average values. This situation is true for both traditional and AIHDA studies and it is an inevitable side-effect in epidemiology. Nevertheless, the perils of stigmatization and false expectations are often ethically justifiable because the aim of the epidemiological analyses is to improve the health and heath care of the individuals and it can to some extent be mitigated via the use of AIHDA. Healthcare policies striving to increase equity always need to take DA into account, in order to identify the group information’s ability to distinguish cases from non-cases. Otherwise, policy interventions intended to improve results for certain groups may prove to be misguided, leaving many exposed individuals without the intervention and many unexposed individuals incorrectly targeted, while possibly also stigmatizing certain groups for being exposed.

One strength of this study is the quality of the data since all information is recorded in a highly standardized way on national registers. The socioeconomic information is recorded by Statistics Sweden with a longstanding experience in the field [53]. Moreover, we can directly compare our results to those presented by the NBHW since the definition of the quality indicator is the same.

### Limitations

PIM among older adults is mainly a process indicator [27]. Process indicators are traditionally evidence-based and measure a segment of a process where the healthcare system is accountable and often regulated by guidelines. In general, process indicators are less susceptible to confounding (i.e., patient mix) than outcome quality indicators. However, measurement of PIM is also susceptible to confounding as on some occasions the existence of PIM is not unwanted but could be therapeutically acceptable on an individual level. Therefore the exact benchmark level of PIM is difficult to define [16]. In a recent study in a Swedish setting [54], it has been concluded that one of seven cases of PIM and potential prescribing omissions (PPO) according to quality indicators EU [7]-PIM list and STOPP/START-criteria, was clinically justified. 63% of the patients with PIM/PPO were assessed as having adequate treatment. Therefore the authors proclaims a cautious approach when evaluating PIM/PPO [54]. This is a validity issue for PIM as a quality indicator, whereas having PIM might in fact not be inappropriate at all. Our study does not evaluate the validity of PIM as a quality indicator, rather we present a way of analysing quality indicators in general, but we want to acknowledge these findings. In this study, we adopted the definition used by the Swedish NBHW, as our aim was to demonstrate how to perform an analysis applying the AIHDA-framework.

Drug dispensations for inpatient care within hospitals and nursing homes from drug storerooms are not included in the SPDR. This could introduce a bias in our results since we do not control for patient mix in the different regions or socioeconomic strata. Groups with higher rates of individuals being cared at hospitals or nursing homes could have PIM that is not registered at the SPDR and therefore these groups’ PIM usage could be underestimated in our results. This limitation also applies to NBHW’s quality indicator.

According to NBHW’s benchmark value, specified as “as low as possible”, we arbitrarily set the target benchmark value for this study equal to the lowest achievable PIM prevalence in a real-world setting in our study (i.e., the prevalence in the strata of 75–79-year-old native men with high income). Another alternative would be to set the benchmark value to observed prevalence levels of PIM in previous studies. However, the definitions of PIM, reported crude prevalence of PIM and benchmark values of PIM varies greatly across different studies [39, 40] and it is therefore less suitable to set the benchmark-value in accordance to studies in other settings.

In our study, we define a case of PIM as having a dispensation of any PIM defined by NBHW (Table 1) anytime during a 12-month period. NBHW uses the same definition of included medications, however it does not use a 12-month period. NBHW’s method for calculating PIM is performed via a special software which analyses the dispensed dose of PIM for every patient and calculates the time covered by each dispensation. A case is then defined as being covered by the last PIM dispensation at a specific point in time, as point prevalence.

Compared to our definition with a 12-month period, NBHW’s method will most likely generate a lower estimate of PIM, since only very large doses or repeated dispensations would count as having PIM for the entire year or more. A small dose on the other hand, would result in only partial use of PIM for that year and that may not coincide with the time point of estimation. Measuring PIM with 12-month prevalence is common in scientific studies [41, 43, 48, 49] and therefore we opted for this definition. We want to describe this departure from NBHW definition if questions arise about why NBHW’s reporting of PIM is considerably lower than our results. When comparing reported prevalence of PIM between different settings (time, place, group of patients) the definitions can greatly affect the results [40]. The criteria which PIM is based on (e.g. Beers, Meds75+, AGS) and the follow-up period are relevant factors. To accurately compare different administrative regions, patient-strata, nationwide results etc., the definitions need to be coherent.

A limitation of this study is the exclusion of 73,245 individuals who either died, emigrated, or had incomplete data on their country of birth, representing approximately 9.1% of the final study population. This exclusion may introduce a selection bias. However, we believe this limitation does not affect our conclusions as the selection criteria align with those used by NBHW. Another limitation is the use of household income as a proxy for socioeconomic position, which may not fully capture socioeconomic position related to financial assets or educational levels. The categorization of country of birth in this study could be refined beyond the binary native and immigrant classification. The term ‘immigrant’ is broad and rather unspecific, including vastly different cultural, economic and educational heritages from different countries. More detailed categories would however lead to a higher number of strata, with less observations within each stratum. Therefore, we chose to include this definition, but other definitions could be explored in future studies. Likewise, the impact of age might be underestimated as we used only three age categories in our analysis. We chose three categories to maintain manageable strata sizes, but for other quality indicators, more age groups may be beneficial.

In AIHDA, AUC is used as a measure of DA. In situations where information bias may result in a higher likelihood of false negatives compared to false positives, such as under-reporting, misclassification, loss to follow-up or poor sensitivity in diagnostic tests, the AUC-value can be misleading if we assign equal importance to both false negatives and false positives. Nonetheless, this issue does not generally affect biomedical applications, nor our specific study, as the identification of positive and negative events (i.e., whether a patient uses PIM or not) is registered-based information from SPDR and the data is less likely to be biased.

The AUC value might also vary depending on the size of the groups under comparison. For example, a large stratum with a high absolute risk of PIM will still have many individuals without PIM, thus reducing the AUC. This issue can be mitigated by weighting the strata inversely based on their size or by employing other discriminatory accuracy metrics that consider components of variance, like the variance partition coefficient [9]. In our analysis, we use unweighted results, which are relevant per se. A low unweighted AUC implies that any interventions to reduce PIM usage should not focus on specific strata but rather be applied universally across all strata.

Conceptually, the MAIHDA approach adopts a multilevel perspective that partitions the individual differences into within groups (level 1) and between groups (level 2). What distinguishes a MAIHDA [6, 9, 10, 14, 34, 52, 55–57] from an AIHDA, is the statistical analysis, not the conceptual idea of how variation in healthcare quality need to be analysed on an individual level and group level simultaneously. From a statistical point of view, this approach can be implemented using both traditional regression analyses with the group as fixed effects [13, 32, 51, 58] or more advanced multilevel regression analyses (MLRA) with a random intercept effect for the groups. Alternatively we could distinguish between ”random effects MAIHDA” or simply “MAIHDA” respectively ”fixed effects MAIHDA” or simply “AIHDA”.

MLRA, provide conceptual and methodological advantages as compared to traditional fixed effects analyses that have been discussed elsewhere [15]. For instance, both traditional and multilevel analyses consider the groups as contexts but in traditional fixed effects analyses all information is modelled at the first level (individual) while when using MLRA the groups are explicitly modelled as a second level random effects. In MLRA the relative differences (e.g., prevalence ratios) have the grand mean (i.e., the mean of the groups means) as reference while in traditional analyses the reference is a specific group (as in our present study). MLRA can better handle groups with few individuals by providing reliability weighted estimations. In addition to the AUC for assessing the importance of the group effects [59], MLRA also provides the variance partition coefficient (VPC) [60, 61]. The VPC quantifies the proportion of outcome variation that lies between groups. Having said that, the simpler AIHDA approach still provides much of the relevant information provided by MAIHDA. Therefore, in the current study we aimed to demonstrate the AIHDA framework expecting to increase the understanding and application of AIHDA in current quantitative evaluation of health care quality and public comparison in health care [62, 63].

### In summary

According to our framework (Table 3) the prevalence of PIM among older adults in Sweden did not reach the desired benchmark value of 19%. In addition, some groups have a higher average risk of PIM than other groups, but the DA of the regional and socioeconomic information was very small. Therefore, any intervention aiming to reach the benchmark value should be universal. Our results suggest that in spite the higher risk of PIM in some groups (e.g. strata including women), the exclusive targeting of interventions to specific groups would be unsuitable as many individuals with PIM are in the groups with lower risk of PIM. In other words, we cannot base decisions on strata’s average risk alone, we also need to consider the DA and the number of cases across the groups. A traditional approach would have identified the existence of gender inequalities as women have a higher average risk of PIM than men. While this conclusion is certainly correct, by applying AIHDA we can also conclude that interventions should be universal due to the low DA, in spite of the higher number of PIM cases among women (almost twice the number of cases compared to men).

We need to clarify that our purpose was not to introduce a new statistical technique. Rather, our aim is to introduce an innovative strategy of analysis. By terming this strategy as “AIHDA” we underlie the importance of focusing on components of individual heterogeneity and discriminatory accuracy and not only on differences between group averages (as it is the traditional approach) [8]. MAIHDA was initially conceptualized for geographical analyses of neighbourhoods and health [8, 10, 12, 64] but can be applied on contexts defined by other criterial like socioeconomic or intersectional strata [6, 9, 10, 34, 52, 55, 57, 65, 66]. As indicated by Evans & Erickson [56] (page 4, left paragraph) the intersectional MAIHDA approach *“is an expansion on Multilevel Analysis of Individual Heterogeneity and Discriminatory Accuracy (MAIHDA) into an intersectional framework. The term “MAIHDA” was coined recently by Merlo 2018* [10], *though the uses of multilevel analysis and measures of discriminatory accuracy to examine variation within and between contexts (such as neighbourhoods) pre-date this term* [12]*”.*

The challenge of this study is double. First, we aim to introduce a strategy of analysis that may discomfort the readers habituated with traditional focus on group mean differences [8]. Second, we aim to present this strategy of analysis in a correct but simplified way which may worry the readers habituated to more complicated epidemiological methods. However, we think the challenge is worthy as it can improve the evaluation of inequalities in routine health care.

## Conclusion

A regular monitoring of geographic and socioeconomic inequalities in quality indicators is fundamental for the evaluation of health care quality. For this purpose, we need to apply state of the art statistical and epidemiological methods. The traditional mean-centric approach based on the analysis of differences between groups averages provides incomplete information that might mislead decision makers in healthcare. As illustrated in our study, AIHDA is not complicated to implement and it provides more nuanced information than traditional comparisons based only on group averages. We therefore propose the application of the AIHDA framework for the quantitative evaluation of healthcare quality when performing geographic and socioeconomic comparisons of quality indicators.

## Supplementary Information

Supplementary Material 1.

## Data Availability

Data are available upon reasonable request. Original data are available from the Swedish National Board of Health and Welfare and Statistics Sweden, after approval of the research project by an Ethical Committee and by the data safety committees of Swedish Authorities.

## Abbreviations

AIHDA: Analysis of individual heterogeneity and discriminatory accuracy
AUC: Area under the curve
DA: Discriminatory accuracy
DDD: Defined daily dose
LISA: The longitudinal integration database for health insurance and labour market studies
MAIHDA: Multilevel analysis of individual heterogeneity and discriminatory accuracy
MLRA: Multilevel regression analyses
NBHW: The Swedish national board of health and welfare
PIM: Potentially inappropriate medication
PIP: Potentially inappropriate prescribing
PPO: Potential prescribing omissions
PR: Prevalence ratio
PRa: Prevalence ratio adjusted
ROC curve: Receiver operating characteristic curve
SALAR: The Swedish association of local authorities and regions
SCB: Statistics Sweden
SPDR: Swedish prescribed drug register
TPR: Swedish total population register
