# Supplementary material for: Geographic and socioeconomic differences in potentially inappropriate medication among older adults – applying a simplified analysis of individual heterogeneity and discriminatory accuracy (AIHDA) for basic comparisons of healthcare quality
Source: BMC Health Serv Res. 2025 Aug 28;25:1144. doi: 10.1186/s12913-025-13335-y (PMC12395699; doi:10.1186/s12913-025-13335-y)
Supplement: Supplementary file 1 — Supplementary Material 1. [file 12913_2025_13335_MOESM1_ESM.docx]

**Supplementary material (SM)**

| SM 1. Number of individuals (n) with potentially inappropriate medication (PIM), total number of individuals (N), and cumulative number of individuals in the five strata with the highest and the lowest prevalences, as well as crude prevalence (P), and prevalence ratios (PR) of PIM during the following 12 months unadjusted and adjusted (PRa) for socioeconomic variables among individuals 75 years and older residing in Sweden by 31 December 2010, grouped into the 21 Swedish administrative regions. Values are numbers, percentages (%) and 95% confidence intervals (CI) | | | | | | | | | | | | |  |
| --- | --- | --- | --- | --- | --- | --- | --- | --- | --- | --- | --- | --- | --- |
|  | |  |  |  | | |  | | |  | |  |  |
| Administrative regions | | n | N | P (95% CI) % | | | PR (95% CI) | | | PRa (95% CI) | |  |  |
| Gävleborg | | *5288* | 25 113 | | 21.06 (20.55-21.56) | | | 0.89 (0.86-0.91) | | | 0.89 (0.87-0.92) | | |
| Värmland | | *5662* | 25 973 | | 21.80 (21.30-22.30) | | | 0.92 (0.90-0.94) | | | 0.93 (0.90-0.95) | | |
| Jämtland | | *2594* | 11 701 | | 22.17 (21.42-22.92) | | | 0.93 (0.90-0.97) | | | 0.94 (0.91-0.98) | | |
| Södermanland | | *5001* | 22 547 | | 22.18 (21.64-22.72) | | | 0.93 (0.91-0.96) | | | 0.94 (0.91-0.96) | | |
| Halland | | *5513* | 24 822 | | 22.21 (21.69-22.73) | | | 0.94 (0.91-0.96) | | | 0.94 (0.92-0.97) | | |
| Östergötland | | 7796 | 34 650 | | 22.50 (22.06-22.94) | | | 0.95 (0.93-0.97) | | | 0.95 (0.93-0.97) | | |
| Kalmar | | 5152 | 22 437 | | 22.96 (22.41-23.51) | | | 0.97 (0.94-0.99) | | | 0.98 (0.95-1.00) | | |
| Uppsala | | 5139 | 22 313 | | 23.03 (22.48-23.58) | | | 0.97 (0.95-1.00) | | | 0.98 (0.95-1.00) | | |
| Västmanland | | 4918 | 21 220 | | 23.18 (22.61-23.74) | | | 0.98 (0.95-1.00) | | | 0.98 (0.95-1.00) | | |
| Örebro | | 5352 | 22 954 | | 23.32 (22.77-23.86) | | | 0.98 (0.96-1.01) | | | 0.98 (0.96-1.01) | | |
| Gotland | | 1173 | 4994 | | 23.49 (22.31-24.66) | | | 0.99 (0.94-1.04) | | | 1.00 (0.95-1.05) | | |
| Dalarna | | 6102 | 25 788 | | 23.66 (23.14-24.18) | | | 1.00 (0.97-1.02) | | | 1.00 (0.98-1.03) | | |
| Skåne | | 22 946 | 96 714 | | 23.73 (23.46-23.99) | | | Reference | | | Reference | | |
| Kronoberg | | 3888 | 16 364 | | 23.76 (23.11-24.41) | | | 1.00 (0.97-1.03) | | | 1.01 (0.98-1.04) | | |
| Stockholm | | 29 392 | 123 235 | | 23.85 (23.61-24.09) | | | 1.01 (0.99-1.02) | | | 0.99 (0.98-1.01) | | |
| Västernorrland | | 5423 | 22 489 | | 24.11 (23.55-24.67) | | | 1.02 (0.99-1.04) | | | 1.03 (1.00-1.05) | | |
| Västerbotten | | 5140 | 21 295 | | 24.14 (23.56-24.71) | | | 1.02 (0.99-1.04) | | | 1.03 (1.00-1.06) | | |
| Norrbotten | | 5400 | 21 959 | | 24.59 (24.02-25.16) | | | 1.04 (1.01-1.06) | | | 1.05 (1.02-1.08) | | |
| Västra Götaland | | 30 762 | 121 930 | | 25.23 (24.99-25.47) | | | 1.06 (1.05-1.08) | | | 1.07 (1.05-1.08) | | |
| Blekinge | | 3762 | 13 947 | | 26.97 (26.24-27.71) | | | 1.14 (1.10-1.17) | | | 1.15 (1.12-1.19) | | |
| Jönköping | | 7908 | 28 894 | | 27.37 (26.85-27.88) | | | 1.15 (1.13-1.18) | | | 1.16 (1.13-1.18) | | |
|  | Area under the ROC curve (AUC) | | | | |  | | | 0.520 | | 0.550 | |  |

| **SM 2.** Number of individuals (n) with potential inappropriate medication (PIM), total number of individuals (N), crude prevalence (P), and cumulative number of individuals in the five strata with the highest and the lowest prevalences, as well as prevalence ratios (PR) of PIM during the following 12 months unadjusted and adjusted (PRa) for intersectional socioeconomic strata among the individuals 75 years and older residing in Sweden by 31 December 2010, grouped into 36 socioeconomic strata. Values are numbers, percentages (%) and 95% confidence intervals (CI). | | | | | | | | | | | |
| --- | --- | --- | --- | --- | --- | --- | --- | --- | --- | --- | --- |
|  | | |  | |  |  | | |  | |  |
| Intersectional socioeconomic strata | | | n | | N | P (95% CI) % | | | PR (95% CI) | | PRa (95% CI) |
| Men |  |  |  | |  |  | | |  | |  |
| 75-79 | High income | Native | 7734 | | 41092 | 18.82 (18.44-19.20) | | | Reference | | Reference |
| 75-79 | Low income | Native | 4843 | | 25143 | 19.26 (18.77-19.75) | | | 1.02 (0.99-1.06) | | 1.02 (0.99-1.05) |
| 75-79 | Middle income | Native | 9490 | | 49162 | 19.30 (18.95-19.65) | | | 1.03 (1.00-1.05) | | 1.02 (1.00-1.05) |
| 75-79 | High income | Immigrant | 704 | | 3513 | 20.04 (18.72-21.36) | | | 1.06 (0.99-1.14) | | 1.07 (1.00-1.14) |
| 80-84 | Low income | Native | 5732 | | 28169 | 20.35 (19.88-20.82) | | | 1.08 (1.05-1.11) | | 1.08 (1.04-1.11) |
| 80-84 | Middle income | Native | 7173 | | 33961 | 21.12 (20.69-21.56) | | | 1.12 (1.09-1.15) | | 1.12 (1.09-1.15) |
| 75-79 | Low income | Immigrant | 1007 | | 4722 | 21.33 (20.16-22.49) | | | 1.13 (1.07-1.20) | | 1.13 (1.07-1.20) |
| 80-84 | High income | Native | 4713 | | 21851 | 21.57 (21.02-22.11) | | | 1.15 (1.11-1.18) | | 1.15 (1.11-1.18) |
| ≥85 | Low income | Native | 5739 | | 26572 | 21.60 (21.10-22.09) | | | 1.15 (1.11-1.18) | | 1.14 (1.11-1.18) |
| 75-79 | Middle income | Immigrant | 1186 | | 5475 | 21.66 (20.57-22.75) | | | 1.15 (1.09-1.22) | | 1.15 (1.09-1.21) |
| 80-84 | Middle income | Immigrant | 658 | | 2999 | 21.94 (20.46-23.42) | | | 1.17 (1.09-1.25) | | 1.17 (1.09-1.25) |
| ≥85 | Middle income | Native | 5421 | | 23881 | 22.70 (22.17-23.23) | | | 1.21 (1.17-1.24) | | 1.20 (1.17-1.24) |
| ≥85 | High income | Native | 3336 | | 14632 | 22.80 (22.12-23.48) | | | 1.21 (1.17-1.26) | | 1.21 (1.17-1.26) |
| 80-84 | Low income | Immigrant | 773 | | 3386 | 22.83 (21.42-24.24) | | | 1.21 (1.14-1.29) | | 1.21 (1.14-1.29) |
| ≥85 | High income | Immigrant | 212 | | 919 | 23.07 (20.34-25.79) | | | 1.23 (1.09-1.38) | | 1.23 (1.09-1.39) |
| ≥85 | Middle income | Immigrant | 373 | | 1602 | 23.28 (21.21-25.35) | | | 1.24 (1.13-1.36) | | 1.24 (1.13-1.36) |
| 80-84 | High income | Immigrant | 412 | | 1745 | 23.61 (21.62-25.60) | | | 1.25 (1.15-1.37) | | 1.26 (1.15-1.37) |
| ≥85 | Low income | Immigrant | 455 | | 1927 | 23.61 (21.72-25.51) | | | 1.25 (1.15-1.36) | | 1.25 (1.16-1.36) |
| Women |  |  |  | |  |  | | |  | |  |
| 75-79 | High income | Native | 8691 | | 36026 | 24.12 (23.68-24.57) | | | 1.28 (1.25-1.32) | | 1.28 (1.25-1.32) |
| 75-79 | High income | Immigrant | 860 | | 3472 | 24.77 (23.33-26.21) | | | 1.32 (1.24-1.40) | | 1.32 (1.24-1.40) |
| 75-79 | Low income | Native | 10554 | | 42278 | 24.96 (24.55-25.38) | | | 1.33 (1.29-1.36) | | 1.32 (1.29-1.36) |
| ≥85 | Low income | Immigrant | 1590 | | 6282 | 25.31 (24.24-26.39) | | | 1.34 (1.28-1.41) | | 1.34 (1.28-1.41) |
| ≥85 | Low income | Native | 15614 | | 61132 | 25.54 (25.20-25.89) | | | 1.36 (1.32-1.39) | | 1.35 (1.32-1.39) |
| 80-84 | Low income | Native | 12073 | | 47002 | 25.69 (25.29-26.08) | | | 1.36 (1.33-1.40) | | 1.36 (1.33-1.40) |
| 75-79 | Middle income | Native | 15823 | | 61554 | 25.71 (25.36-26.05) | | | 1.37 (1.33-1.40) | | 1.36 (1.33-1.40) |
| ≥85 | High income | Native | 4860 | | 18885 | 25.73 (25.11-26.36) | | | 1.37 (1.32-1.41) | | 1.37 (1.33-1.41) |
| 80-84 | High income | Native | 6137 | | 23396 | 26.23 (25.67-26.79) | | | 1.39 (1.35-1.44) | | 1.39 (1.35-1.44) |
| 80-84 | Low income | Immigrant | 1960 | | 7464 | 26.26 (25.26-27.26) | | | 1.40 (1.34-1.46) | | 1.39 (1.33-1.45) |
| ≥85 | High income | Immigrant | 365 | | 1381 | 26.43 (24.10-28.76) | | | 1.40 (1.28-1.54) | | 1.41 (1.29-1.55) |
| 75-79 | Low income | Immigrant | 2228 | | 8341 | 26.71 (25.76-27.66) | | | 1.42 (1.36-1.48) | | 1.42 (1.36-1.47) |
| 80-84 | Middle income | Native | 13592 | | 50284 | 27.03 (26.64-27.42) | | | 1.44 (1.40-1.47) | | 1.43 (1.40-1.47) |
| ≥85 | Middle income | Native | 14008 | | 51655 | 27.12 (26.73-27.50) | | | 1.44 (1.41-1.48) | | 1.44 (1.40-1.48) |
| 80-84 | High income | Immigrant | 610 | | 2213 | 27.56 (25.70-29.43) | | | 1.46 (1.36-1.57) | | 1.47 (1.37-1.58) |
| ≥85 | Middle income | Immigrant | 1129 | | 4081 | 27.66 (26.29-29.04) | | | 1.47 (1.39-1.55) | | 1.47 (1.39-1.55) |
| 75-79 | Middle income | Immigrant | 2462 | | 8881 | 27.72 (26.79-28.65) | | | 1.47 (1.42-1.53) | | 1.47 (1.41-1.53) |
| 80-84 | Middle income | Immigrant | 1794 | | 6261 | 28.65 (27.53-29.77) | | | 1.52 (1.46-1.59) | | 1.52 (1.46-1.59) |
| Area under the ROC curve (AUC) | | | |  |  | |  |  | 0.544 | 0.550 | |

**SM 3.** Prevalence Ratios (PR) and (95% CI) for use of potentially inappropriate medication (PIM) during the following 12 months among the individuals 75 years and older residing in Sweden by 31 December 2010

|  | Sex | Income | Age | Country of birth | All | Multicategory strata | Multicategory strata  and region |
| --- | --- | --- | --- | --- | --- | --- | --- |
| Sex |  |  |  |  |  |  |  |
| Men | Reference |  |  |  | Reference |  |  |
| Women | 1.26 (1.25-1.27) |  |  |  | 1.25 (1.24-1.27) |  |  |
| Income |  |  |  |  |  |  |  |
| Low |  | Reference |  |  | Reference |  |  |
| Middle |  | 1.02 (1.01-1.03) |  |  | 1.04 (1.03-1.05) |  |  |
| High |  | 0.96 (0.95-0.97) |  |  | 1.01 (0.99-1.02) |  |  |
| Age |  |  |  |  |  |  |  |
| 75-79 |  |  | Reference |  | Reference |  |  |
| 80-84 |  |  | 1.07 (1.06-1.09) |  | 1.07 (1.05-1.08) |  |  |
| ≥85 |  |  | 1.10 (1.09-1.11) |  | 1.08 (1.07-1.09) |  |  |
| Country of birth | |  |  |  |  |  |  |
| Native |  |  |  | Reference | Reference |  |  |
| Immigrant |  |  |  | 1.06 (1.05-1.08) | 1.06 (1.04-1.07) |  |  |
| AUC* | 0.535 (0.534-0.536) | 0.509 (0.507-0.510) | 0.515 (0.513-0.516) | 0.504 (0.503-0.505) | 0.543 (0.541-0.544) | 0.544 (0.542-0.545) | 0.550 (0.548-0.551) |
|  | | | |  |  |  |  |
| *Area under the ROC curve | |  |  |  |  |  |  |
